# Supplementary material for: Gene body methylation evolves during the sustained loss of parental care in the burying beetle
Source: Nat Commun. 2024 Aug 4;15:6606. doi: 10.1038/s41467-024-50359-0 (PMC11298552; doi:10.1038/s41467-024-50359-0)
Supplement: Supplementary file 1 — Supplementary Info [file 41467_2024_50359_MOESM1_ESM.pdf]

Supplementary Information

Sarkies *et al.,* (2024) Evolved changes in DNA methylation in response to the sustained loss of parental care in the burying beetle.

Supplementary Note

CpG methylation in the burying beetle

We sought to characterise the methylomes of first instar burying beetle larvae. We found average 5mC across the whole genome to be between 0.7-0.8% for all samples. Average levels of methylation were significantly higher in gene bodies (3.64 ± 8.60) compared to coding sequences (CDS; 2.72 ± 10.8), introns (0.952 ± 4.44), repeat/TEs (0.523 ± 1.96) and upstream regions (5' UTR; 0.659 ± 3.22; all FDR-corrected Wilcoxon *p* values < 2.2×10^{-16}; Figure S2a). Moreover, we found that 5mC levels in gene bodies were weakly but positively correlated with gene expression ($t(1,11920)=36.34$, $p<2.2\times10^{-16}$, R^2 = 0.10). When we classified genes into expression state categories (silent, low, medium or highly expressed; Fig S1a), we found that this correlation was mainly driven by expression states that were classified as medium or low. Medium and high levels of expression did not have significantly different 5mC values from each other (Figure S1b, S2b). These findings are consistent with previous reports of 5mC residing primarily in gene bodies of moderate to highly expressed genes in plants and other insects (23,25,38).

Next, we determined whether the removal of care resulted in changes in 5mC levels at individual CpGs across specific genomic features. We looked for enrichment of differentially methylated CpGs over the expected random distribution of methylated CpGs across genomic features (Figure S2b). A CpG was classified as being methylated if the logit probability of it being methylated was greater than the bisulfite conversion error rate in at least 1 sample (see Methods). The density of methylated CpGs was higher in gene bodies (Figure S2c; $X^2(5, n=7400) = 14786$, $p<2.2\times10^{-16}$) and we found differentially methylated CpGs (in both environmental and evolved contrasts) to be significantly enriched in genes and, specifically, introns (FDR-corrected *p*'s<0.05; Figure S2c) but not in any other annotated features tested.

Supplementary Figures

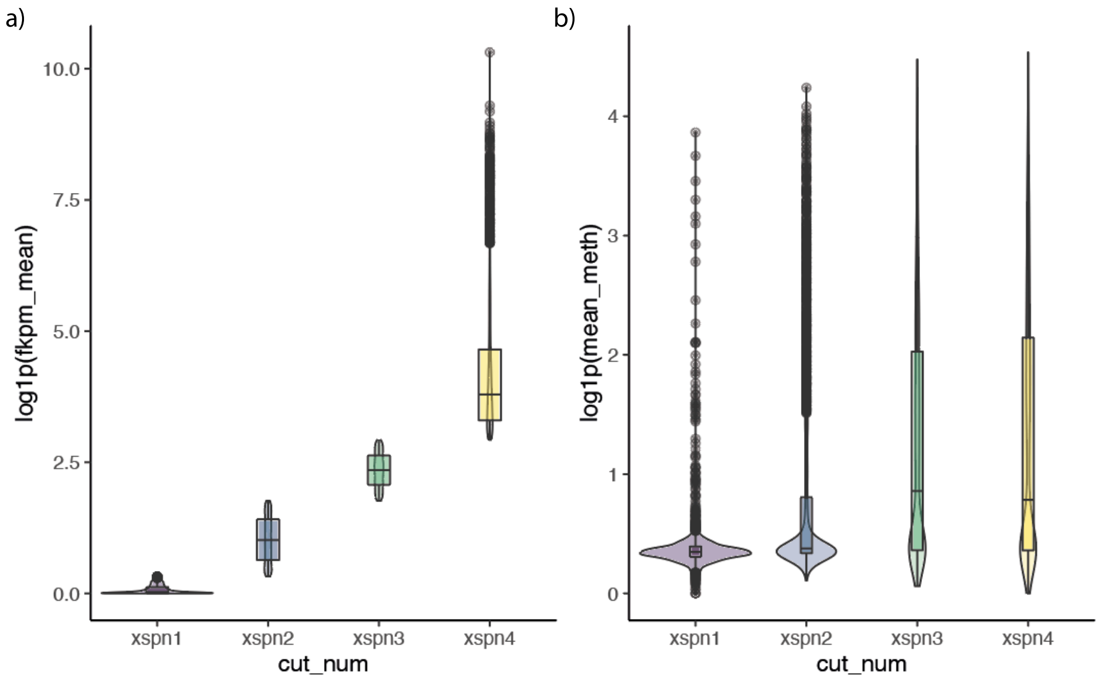

Figure S1. Relationship between gene expression and levels of 5mC of gene bodies. a) Violin plots with box plots showing average gene expression (FKPM) in genes categorised as being inactive (xspn1, n=2981) to low (xspn2, n=2980), intermediate (xspn3, n=2980) and high (xspn4; n=2981) levels of transcriptional activity. **b)** Violin plots with box plots showing average gene body CpG methylation (mCpG/CpG) for genes in differential transcriptional states.

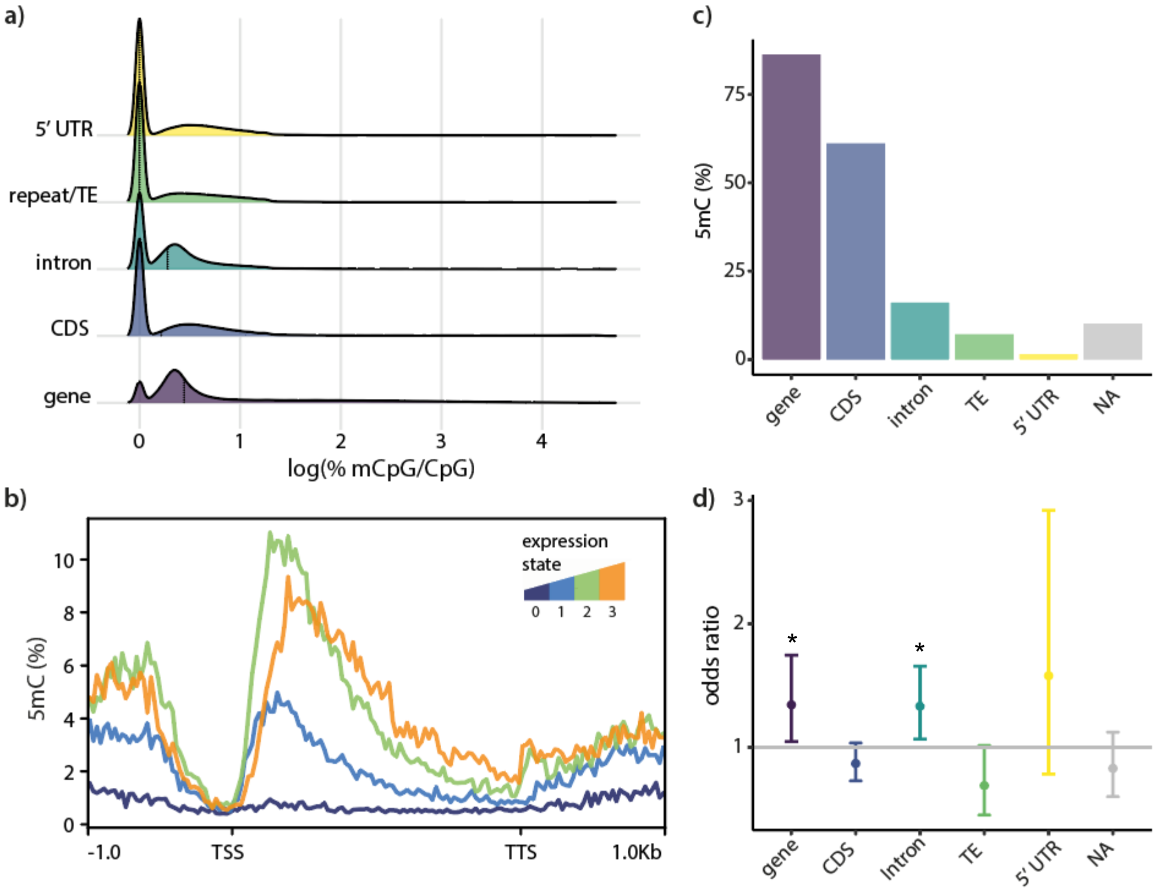

Figure S2. CpG methylation in the burying beetle. a) Ridge plots showing the distribution of percent methylated CpG levels (5mC %) in different genomic features: 1000bp upstream of genes (5' UTR), repeat or transposable elements (TE), introns, coding sequences (CDS) and across the entire gene body (gene). Median 5mC values are indicated by vertical dotted lines. **b)** Average 5mC levels across gene bodies (± 1kb) of genes categories by transcriptional activity: from inactive (0) to genes showing low (1), intermediate (2) and high (3) levels of expression. Plotted regions include 1kb upstream and downstream of transcription start sites (TSS) and transcription termination sites (TTS), respectively for each gene **c)** Bar plot indicating the proportion of methylated CpGs across gene features. **d)** Enrichment scores (odds-ratio; $\log_2$(OR) and 95% confidence intervals) for differentially methylated CpGs across different gene features. (Fishers exact tests; * indicates FDR-corrected $p<0.05$**).**

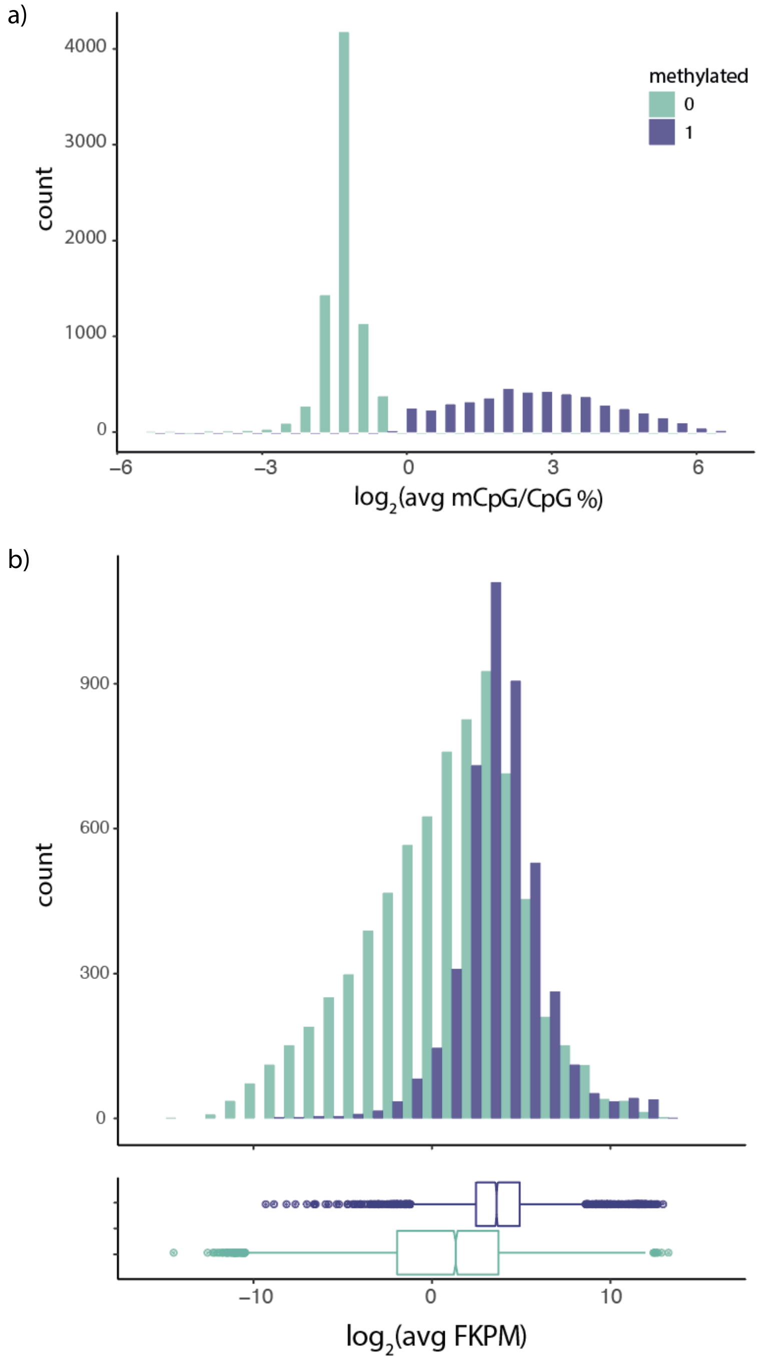

Figure S3. Classification of methylated genes. a) Histogram of methylation (mCpG/CpG) values for genes categorised as methylated (n=4431) or not (n=7491). **b)** histogram (top) and boxplot (bottom) of expression values (FKPM) for genes categorised as being methylated or not.

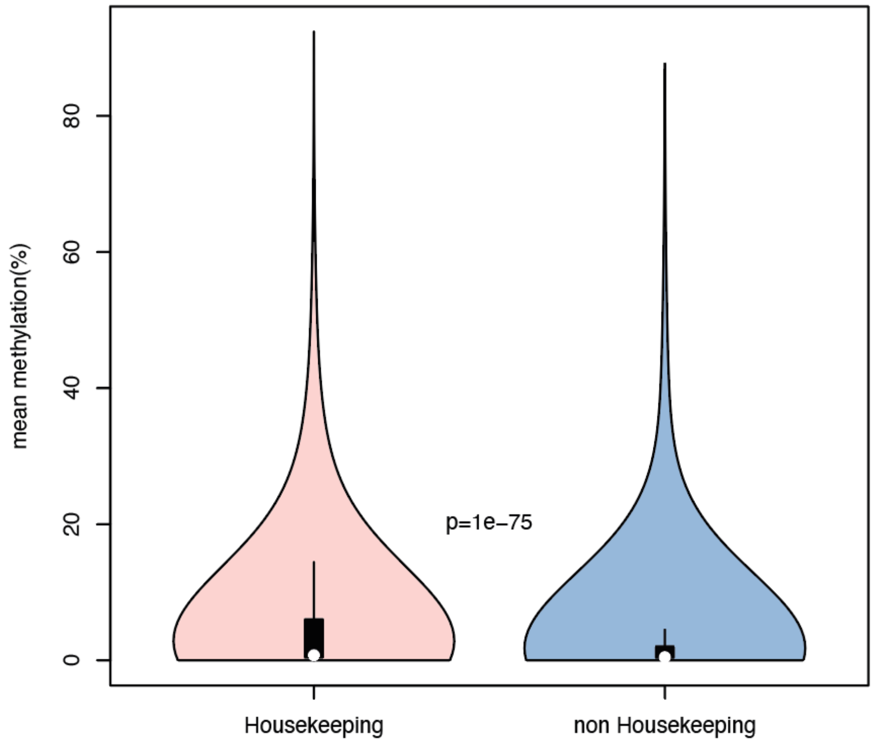

Figure S4. Relationship between 5mC levels and housekeeping genes. Violin plots of mean methylation (mCG/mCpG %) of methylated genes classified as housekeeping (n=2000) and non-housekeeping genes (n=2431). Data are presented as violin plots. White circles represent the median. The lower and upper edges of the boxplot (black) represent the interquartile range. Whiskers extend between the highest and the lowest data points within 1.5 times the interquartile range from the median. The width of the plot illustrates the kernal probability density (density of data points).

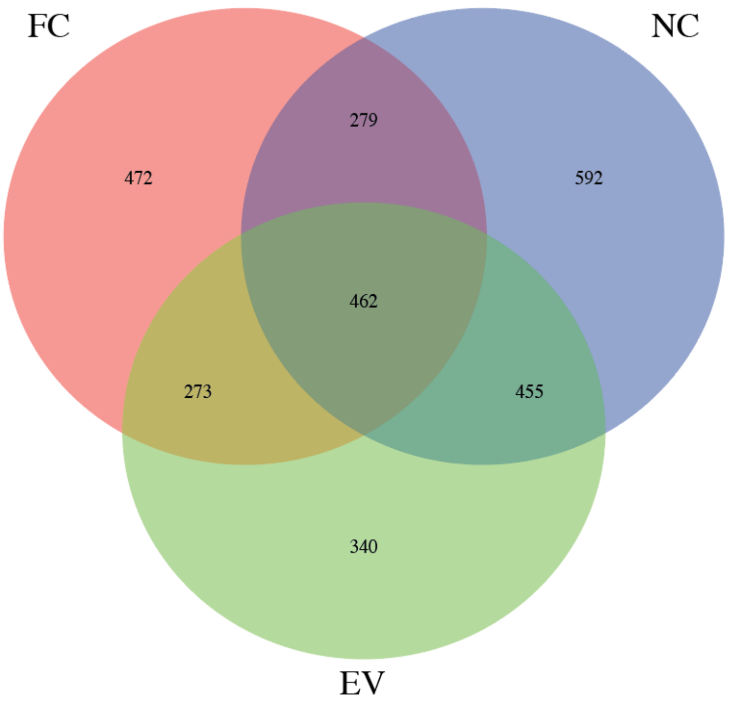

Figure S5. Overlap in differentially methylated genes across the different conditions. Venn diagrams indicating overlap in DMGs across the three contrasts analysed: Evolved (EV; population effect NC_{POP} vs FC_{POP} irrespective of current environment; green), effect of the NC environment within the FC population (FC; $FC_{POP}FC_{ENV}$ vs $FC_{POP}NC_{ENV}$; red) and effect of the NC environment within the NC population (NC; $NC_{POP}FC_{ENV}$ vs $NC_{POP}NC_{ENV}$; blue).

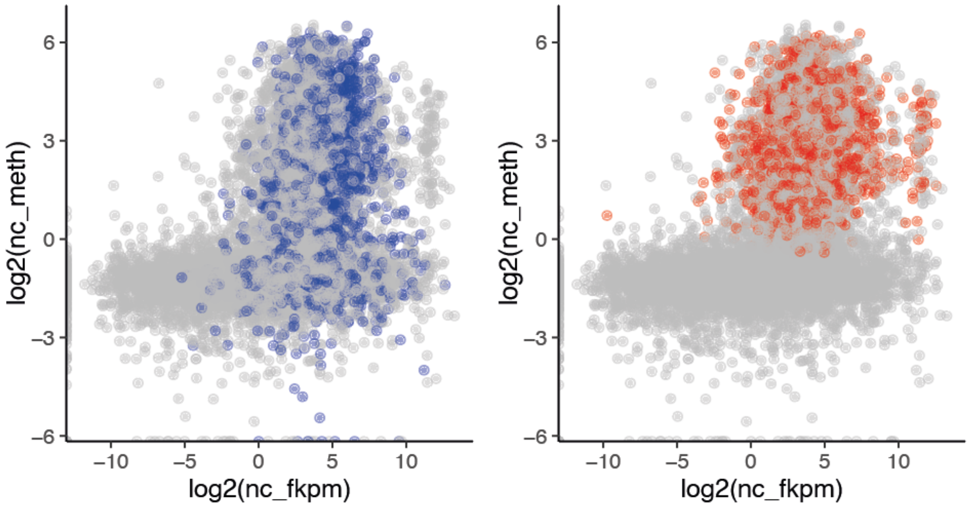

Figure S6. Relationship between expression and methylation. The relationship between gene expression (FKPM) and percent CpG methylation (mCpG/CpG) for all genes analysed in the response to a NC_{ENV} versus FC_{ENV} within the NC_{POP}. Colours indicated differentially expressed genes (DEGs; blue; left panel) and differentially methylated genes (DMGs; purple; right panel) for each contrast. See methods for how DMGs and DEGs were determined.

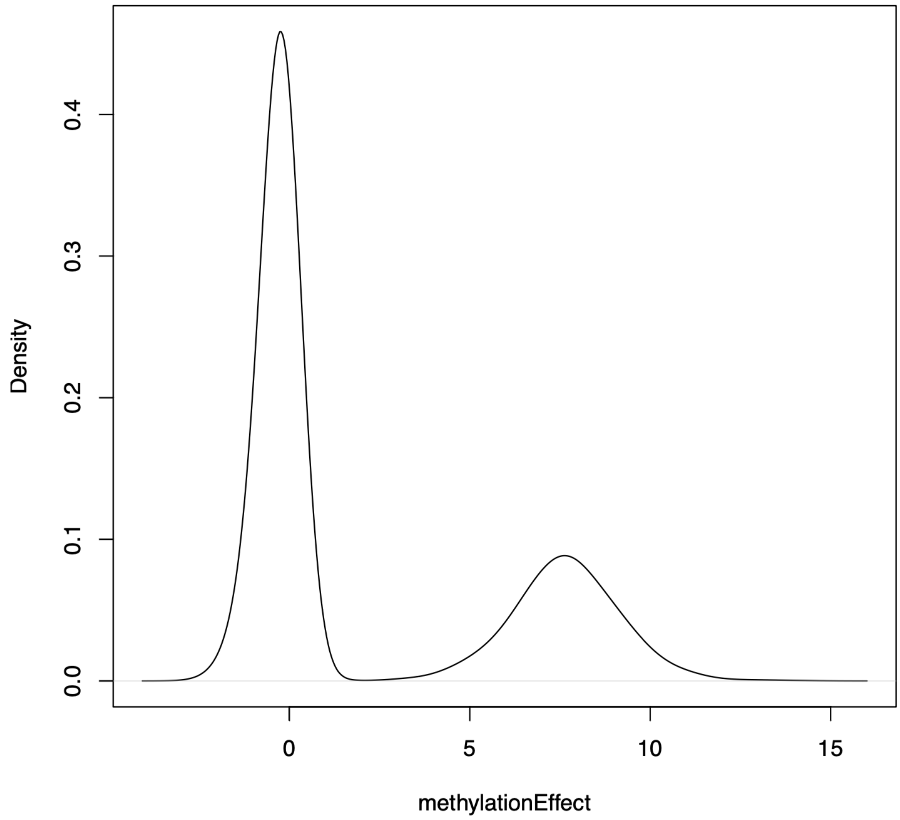

Figure S7. Using random effect structure of genes to classify methylated genes. Density plot of random effect calculation (see Methods) showing methylated (right peak) and unmethylated (left peak) genes for $FC_{POP}FC_{ENV}$.

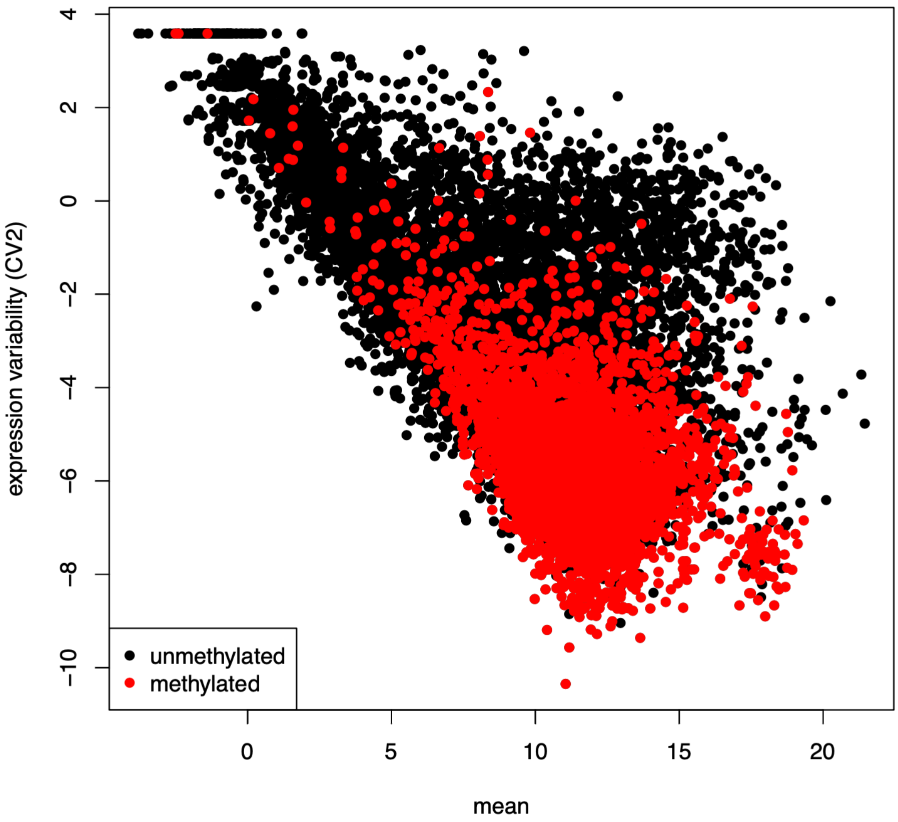

Figure S8. Relationship between mean gene expression and expression variability. Plot showing relationship between coefficient of variation (CV^2) and mean expression (counts) for each gene. Red points indicate genes that are methylated, black points indicated unmethylated genes.

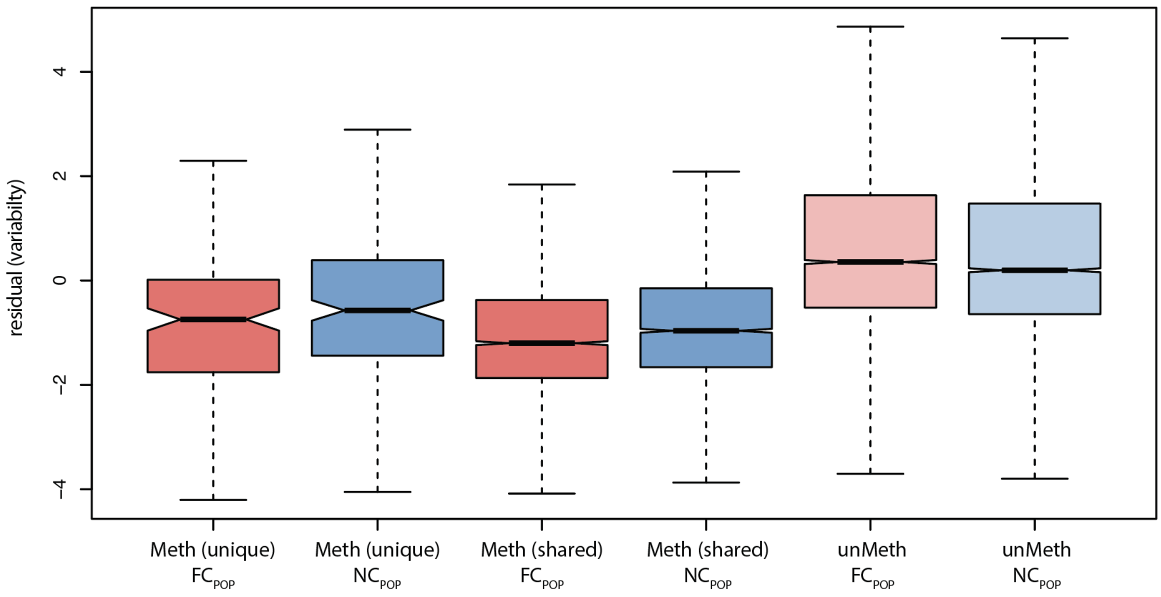

Figure S9. Relationship between methylated genes and gene expression variability. Gene expression variability (residuals from loess fit; see Methods) was significantly reduced (all p's < 2.2e-16) in uniquely (FC_{POP} n=171, NC_{POP} n=217 and shared methylated (Meth) genes compared to unmethylated (unMeth) genes in both Full Care (FC_{POP}) and No Care (NC_{POP}) population (two-sided Wilcoxon test). Data are presented as boxplots. Horizontal lines (bold and black) represent the median. The lower and upper edges of the boxplot represent the interquartile range. Whiskers extend between the highest and the lowest data points within 1.5 times the interquartile range from the median.

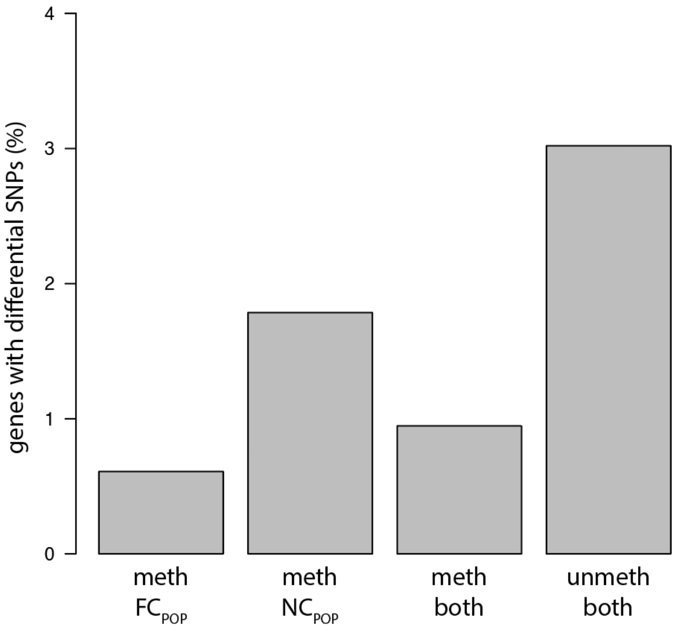

Figure S10. Small nucleotide polymorphisms (SNPs) in methylated genes. Percent of 5mC genes containing differential SNPs between populations as a function of methylated genes unique to each population (meth FC_{POP} or meth NC_{POP}) compared to genes that were methylated (meth) or not methylated (unmeth) in. Genes with 5mC in general showed a reduced tendency to contain highly differentiated SNPs compared to genes that were not methylated in either population (Fisher's test, $p=1x10^{-15}$).
