# Supplementary material for: Gene body methylation evolves during the sustained loss of parental care in the burying beetle
Source: Nat Commun. 2024 Aug 4;15:6606. doi: 10.1038/s41467-024-50359-0 (PMC11298552; doi:10.1038/s41467-024-50359-0)
Supplement: Supplementary file 3 — Reporting summary [file 41467_2024_50359_MOESM3_ESM.pdf]

Corresponding author(s): Mashoodh/Kilner

Last updated by author(s): Jan 25, 2024

Reporting Summary

Nature Portfolio wishes to improve the reproducibility of the work that we publish. This form provides structure for consistency and transparency in reporting. For further information on Nature Portfolio policies, see our Editorial Policies and the Editorial Policy Checklist.

Statistics

For all statistical analyses, confirm that the following items are present in the figure legend, table legend, main text, or Methods section.

n/a	Confirmed	
☐	☒	The exact sample size (*n*) for each experimental group/condition, given as a discrete number and unit of measurement
☐	☒	A statement on whether measurements were taken from distinct samples or whether the same sample was measured repeatedly
☐	☒	The statistical test(s) used AND whether they are one- or two-sided<br>*Only common tests should be described solely by name; describe more complex techniques in the Methods section.*
☐	☒	A description of all covariates tested
☐	☒	A description of any assumptions or corrections, such as tests of normality and adjustment for multiple comparisons
☐	☒	A full description of the statistical parameters including central tendency (e.g. means) or other basic estimates (e.g. regression coefficient) AND variation (e.g. standard deviation) or associated estimates of uncertainty (e.g. confidence intervals)
☐	☒	For null hypothesis testing, the test statistic (e.g. *F*, *t*, *r*) with confidence intervals, effect sizes, degrees of freedom and *P* value noted<br>*Give P values as exact values whenever suitable.*
☐	☒	For Bayesian analysis, information on the choice of priors and Markov chain Monte Carlo settings
☐	☒	For hierarchical and complex designs, identification of the appropriate level for tests and full reporting of outcomes
☐	☒	Estimates of effect sizes (e.g. Cohen's *d*, Pearson's *r*), indicating how they were calculated

Our web collection on statistics for biologists contains articles on many of the points above.

Software and code

Policy information about availability of computer code

Data collection	N/A
Data analysis	Bioinformatic processing: TrimGalore (0.5.0), HiSat2 (2.1.0),FastQC (0.11.9), Stringtie (2.0.3), BISCUIT (1.0.0), RepeatModeller (2.0.1), RepeatMasker (4.1.0), Bedtools (2.29.2), BLAST2GO (5.1.1) and Bismark (0.22.3). Stastical analyses: R version (4.1.2) and R packages: tidyverse (2.0.0), DESeq2 (1.26.0), MCMCglmm (2.29), topGO (2.38.1), mixtools (2.0.0), lme4 (1,1-34) and adegenet (2.1.3).

For manuscripts utilizing custom algorithms or software that are central to the research but not yet described in published literature, software must be made available to editors and reviewers. We strongly encourage code deposition in a community repository (e.g. GitHub). See the Nature Portfolio guidelines for submitting code & software for further information.

Data

Policy information about availability of data

All manuscripts must include a data availability statement. This statement should provide the following information, where applicable:

- Accession codes, unique identifiers, or web links for publicly available datasets
- A description of any restrictions on data availability
- For clinical datasets or third party data, please ensure that the statement adheres to our policy

All raw sequencing data generated by this study have been submitted to the NCBI Gene Expression Omnibus (GEO) under accession number (GSE171776).

Research involving human participants, their data, or biological material

Policy information about studies with human participants or human data. See also policy information about sex, gender (identity/presentation), and sexual orientation and race, ethnicity and racism.

Reporting on sex and gender	N/A
Reporting on race, ethnicity, or other socially relevant groupings	N/A
Population characteristics	N/A
Recruitment	N/A
Ethics oversight	N/A

Note that full information on the approval of the study protocol must also be provided in the manuscript.

Field-specific reporting

Please select the one below that is the best fit for your research. If you are not sure, read the appropriate sections before making your selection.

☐ Life sciences ☐ Behavioural & social sciences ☒ Ecological, evolutionary & environmental sciences

For a reference copy of the document with all sections, see nature.com/documents/nr-reporting-summary-flat.pdf

Ecological, evolutionary & environmental sciences study design

All studies must disclose on these points even when the disclosure is negative.

Study description	Wild-caught population were exposed to care (FC) or no care (NC) for 30 generations. Each treatment was replicated twice and comprised of 50-75 families at each generation. Source population and breeding data described in Schraeder et al., 2017 and Mashoodh et al., 2023. RNA and DNA methylation (5mC) was analysed in larvae on the 30th generation.
Research sample	For molecular analyses we sampled multiple larvae from 15 families on generation 30 from each population replicate. Half were in their native condition (either FC in FC or NC in NC) or half were in their reciprocal condition (e.g., FC in NC or NC in FC). For a total of 4 conditions: FCFC, NCNC, FCNC and NCNC. From this we created sequencing libraries by pooling larvae for RNA-seq (11-12 libraries/condition; 4 larvae per library) and bisulfite-sequencing libraries (3-4 libraries/condition; 8-11 larvae per library).
Sampling strategy	Unrelated families were randomly selected. Sample sizes were chosen based on standards in the field for both methylation and RNA analyses and increasing pooling of multiple individuals to increase finding results of large effect. This strategy is similar to other experimental evolution studies conducted in other insects (e.g., Drosophila).
Data collection	Animal husbandry and breeding data was collected by several students, postdocs and research assistants over the course of experimental evolution and recorded on physical datasheets and recorded in shared excel sheets. Samples were collected and snap frozen by PI Mashoodh with the help of students (acknowledged in manuscript).
Timing and spatial scale	Experimental evolution begain in 2014. Samples were collected in 2018 and flash frozen for subsequent analyses.
Data exclusions	All data was retained except for RNA sequencing where lowly expressed genes (those with less than 15 counts in more than 90% of samples) were filtered from raw counts table.
Reproducibility	We have replicated the experimental evolution across two blocks per experimental condition. Previous work has shown concordance between blocks (Mashoodh et al.)
Randomization	Wild-caught individuals were bred in the lab. From each family, Siblings were randomly assigned across the 4 populations to ensure that each replicate line was genetically identical and divergence could be attributed to the expeirmental manipulation. Families selected for molecular analyses were randomly selected and were unrelated (within population) to maximise population level inference. Individual larvae were balanced across libraries to sample block structure and families equally across the libraries.
Blinding	During breeding experimenters were not blind to conditions as care needed to be manipulated and selection lines needed to be kept separately to prevent accidental interbreeding. Upon sample collection, samples were assigned unique IDs that made experimenters blind to experimental condition.

Did the study involve field work? ☐ Yes ☒ No

Reporting for specific materials, systems and methods

We require information from authors about some types of materials, experimental systems and methods used in many studies. Here, indicate whether each material, system or method listed is relevant to your study. If you are not sure if a list item applies to your research, read the appropriate section before selecting a response.

Materials & experimental systems

n/a	Involved in the study
☒	☐ Antibodies
☒	☐ Eukaryotic cell lines
☒	☐ Palaeontology and archaeology
☐	☒ Animals and other organisms
☒	☐ Clinical data
☒	☐ Dual use research of concern
☒	☐ Plants

Methods

n/a	Involved in the study
☒	☐ ChIP-seq
☒	☐ Flow cytometry
☒	☐ MRI-based neuroimaging

Animals and other research organisms

Policy information about studies involving animals; ARRIVE guidelines recommended for reporting animal research, and Sex and Gender in Research

Laboratory animals	Populations of Nicrophorus vespilloides that were maintained in the lab across 30 generations.
Wild animals	This particular study does not report on wild animals, though source populations were based on wild-caught beetles (permission granted from local Woodland Trusts)
Reporting on sex	N/A
Field-collected samples	N/A
Ethics oversight	Ethical oversight is currently not required for the use of arthropods for scientific purposes in the UK.

Note that full information on the approval of the study protocol must also be provided in the manuscript.

Plants

Seed stocks	*Report on the source of all seed stocks or other plant material used. If applicable, state the seed stock centre and catalogue number. If plant specimens were collected from the field, describe the collection location, date and sampling procedures.*
Novel plant genotypes	*Describe the methods by which all novel plant genotypes were produced. This includes those generated by transgenic approaches, gene editing, chemical/radiation-based mutagenesis and hybridization. For transgenic lines, describe the transformation method, the number of independent lines analyzed and the generation upon which experiments were performed. For gene-edited lines, describe the editor used, the endogenous sequence targeted for editing, the targeting guide RNA sequence (if applicable) and how the editor was applied.*
Authentication	*Describe any authentication procedures for each seed stock used or novel genotype generated. Describe any experiments used to assess the effect of a mutation and, where applicable, how potential secondary effects (e.g. second site T-DNA insertions, mosiacism, off-target gene editing) were examined.*
