# Supplementary material for: Gene body methylation evolves during the sustained loss of parental care in the burying beetle
Source: Nat Commun. 2024 Aug 4;15:6606. doi: 10.1038/s41467-024-50359-0 (PMC11298552; doi:10.1038/s41467-024-50359-0)
Supplement: Supplementary file 4 — Description Of Additional Supplementary File [file 41467_2024_50359_MOESM4_ESM.pdf]

Description of Additional supplementary files

Supplementary Data 1

Description: Table of differentially expressed genes unique to the No Care population (NCPOP) with base mean expression (baseMean), log-fold change (log2FoldChange) 4and error (lfcSE), p-value and adjusted p-value (padj).

Supplementary Data 2

Description: GO enrichment result tables for differentially expressed and differentially methylated genes for environmental (exposure to No Care) versus evolved changes 1 (sustained exposure to No Care).
